# Book Reviews

**Published:** 1903-02

**Authors:** 


### BOOK REVIEWS.

CULBRETH'S MATERIA MEDICA AND PHARMACOLOGY. Third Edition. A Manual of Materia Medica and Pharmacology, comprising all Organic and Inorganic Drugs which are and have been official in the United States Pharmacopœia, together with Allied Species and Useful Synthetics. By David M. R. Culbreth, M.D., Professor of Botany, Materia Medica and Pharmacognosy in the Maryland College of Pharmacy; Professor of Materia Medica and Pharmacognosy in the University of Maryland Medical and Dental Schools, Baltimore. Third edition enlarged and thoroughly revised. In one octavo volume of 905 pages, with 473 illustrations, cloth, $4.75, net. Lea Brothers & Co., Publishers; Philadelphia and New York, 1903.

The popularity of this book is attested by the fact that the second edition more than doubled the sales of the former, and that a third edition was so soon demanded. The last edition is thoroughly revised and foreshadows the new U. S. Pharmacopœia. The revision of this work takes in the official list of drugs admitted by the pharmacopœia. The work is arranged on the basis of associating those substances with a common or allied origin, allowing those next related to follow in regular order. Measurements are expressed in the ordinary and metric scales, and temperature is stated in both Fahrenheit and Centigrade. There are pages devoted to the proper pronunciation of difficult words. The illustrations are numerous, and quite a number of new ones have been added. Nothing has been spared to make this a complete work of its kind.

---

THE A-B-C OF PHOTO-MICROGRAPHY. A Practical Hand-Book for Beginners. By W. H. Walmsley. 155 pages, 5x7, with 29 photo-micrographs by the author. Cloth, $1.25, net. Tennant & Ward, New York.

The fascinating subject of photo-micrography is dealt with in this small work in a plain but comprehensive manner. Any one who desires information on this subject will do well to procure the book. He will find with its assistance that the technic and difficulties are easily mastered, and the results well repay him for his trouble.

---

A POCKET TEXT-BOOK OF ANATOMY. By Wm. H. Rockwell, Jr., M.D., Assistant Demonstrator of Anatomy, College of Physicians, Columbia University, New York. In one 12mo volume of 600 pages, with 70 illustrations. Lea's Series of Pocket Text-Books. Edited by Bern B. Gallaudet, M.D. Cloth, $2.25, net; Limp Leather, $2.75, net. Lea Brothers & Co., Philadelphia and New York.

This book belongs to the Pocket Text-Book series. It is not intended to take the place of the more elaborate works on the subject, but is designed to give those facts in anatomy which are essential to the study of the subject. As such and as a safe and competent guide for the student it will prove of service.

---

### CREMATORIES.

The first crematory in the United States was established in Washington, Pa., in 1876. In spite of prejudice of custom, cremation is making such rapid headway that there are now 200 crematories scattered over the country from the Atlantic to the Pacific. In New York the Fresh Pond Crematory was built in 1885, and during that year only eight bodies were incinerated. Last year the incinerations numbered 654. Among the clergy of every denomination, especially in the Episcopal church, who favor this disposal of the dead may be mentioned Bishop Potter, Bishop Lawrence, Rev. Dr. Rainsford, Dean Hodges and Rev. Wm. R. Huntington. Some of the more advanced of the Catholic clergy also favor cremation.

